# Efficacy and Safety of Intraosseous Regional Versus Intravenous Tranexamic Acid in Total Knee Arthroplasty: A Randomized Controlled Trial

**DOI:** 10.2106/JBJS.OA.26.00282

**Published:** 2026-10-15

**Authors:** Shuaifei Tian, Baochao Ji, Yicheng Li, Xiaogang Zhang, Guoqing Li, Boyong Xu, Li Cao

**Affiliations:** 1 Department of Orthopaedics, First Affiliated Hospital of Xinjiang Medical University, Ürümqi, Xinjiang, China; 2 Key Laboratory of High Incidence Disease Research in Xingjiang (Xinjiang Medical University), Ministry of Education, Urumqi, China; 3 Xinjiang Clinical Research Center for Orthopedics, Urumqi, China

## Abstract

**Background:**

Tranexamic acid (TXA) reduces perioperative blood loss in total knee arthroplasty (TKA); however, evidence for intraosseous regional administration (IORA) remains limited. We evaluated the efficacy and safety of IORA-TXA versus intravenous TXA (IV-TXA) in TKA.

**Methods:**

In this prospective, randomized, single-center trial, 98 patients undergoing primary TKA were randomized 1:1 to IORA-TXA or IV-TXA (49 per group) at a uniform dose of 20 mg/kg. The primary outcome was calculated perioperative blood loss. Secondary efficacy outcomes included calculated hidden blood loss, postoperative hemoglobin (Hb) levels, and transfusion rate. Safety outcomes included deep vein thrombosis (DVT), muscular calf vein thrombosis, pulmonary embolism (PE), and wound-related complications.

**Results:**

Compared with IV-TXA, IORA-TXA reduced calculated perioperative blood loss (674.8 ± 335.0 vs. 817.5 ± 357.2 mL; mean difference, 142.6 mL; 95% CI, 7.4-277.8; p = 0.038) and calculated hidden blood loss (644.8 ± 335.5 vs. 790.9 ± 339.0 mL; mean difference, 146.1 mL; 95% CI, 10.9-281.3; p = 0.035). Postoperative Hb was similar on POD1 (134.0 ± 9.8 vs. 130.1 ± 11.1 g/L; p = 0.067) but was higher with IORA-TXA on POD2 (119.8 ± 8.3 vs. 113.8 ± 11.5 g/L; mean difference 6.0 g/L; 95% CI 2.0-10.0; p = 0.005) and POD3 (114.8 ± 7.9 vs. 109.8 ± 12.6 g/L; mean difference 5.0 g/L; 95% CI 0.8-9.2; p = 0.020). No patient required blood transfusion. DVT detected by routine postoperative Doppler occurred numerically more often with IORA-TXA than with IV-TXA: 10.2% (95% CI, 3.4%-22.2%) versus 0.0% (95% CI, 0.0%-7.3%); p = 0.056.

**Conclusions:**

IORA-TXA reduced calculated perioperative and hidden blood loss and improved early postoperative Hb preservation compared with IV-TXA. However, DVT incidence was numerically higher with IORA-TXA, indicating a potential thromboembolic safety signal that warrants confirmation in larger, adequately powered trials.

**Level of Evidence:**

Therapeutic Level I. See Instructions for Authors for a complete description of levels of evidence.

## Introduction

Total knee arthroplasty (TKA) is the definitive surgical treatment of end-stage knee osteoarthritis, but perioperative blood loss remains problematic, contributing to anemia, transfusion, and delayed recovery^1,2^. Intravenous (IV) tranexamic acid (TXA) reduces drainage and transfusion in TKA^3,4^, yet systemic administration may provide limited intra-articular exposure and transient hemostasis^5,6^. Venous thromboembolism concerns persist in higher-risk patients, although evidence remains controversial.

Intraosseous regional administration (IORA) enables targeted drug delivery to the operative limb under tourniquet control. By exploiting the intraosseous venous plexus, this technique can produce high distal limb tissue concentrations while limiting systemic exposure^7-10^. Initially developed for antibiotics and later extended to analgesics and nonsteroidal anti-inflammatory drugs^11,12^, IORA may serve as a regional pharmacotherapeutic platform. Applied to TXA, IORA could enhance local antifibrinolytic activity while reducing systemic exposure. Although preclinical studies suggest comparable systemic availability between intraosseous and intravenous administration^13-16^, the clinical efficacy and safety of IORA-TXA in total knee arthroplasty remain uncertain.

Therefore, this study evaluated the efficacy and safety of IORA-TXA in TKA. Through this study, we aim to provide a new, more targeted perioperative hemostatic strategy to enhance patient recovery.

## Materials and Methods

### Study Design and Patients

This prospective, randomized, single-center trial was conducted in the orthopaedics department of the First Affiliated Hospital of Xinjiang Medical University. The study followed the Declaration of Helsinki and CONSORT guidelines^17^, was registered with the Chinese Clinical Trial Registry (CTR2400086474), and received institutional ethics approval. Written informed consent was obtained before enrollment.

Patients were recruited between July 2024 and July 2025 by a research investigator not involved in surgery. Eligible patients were aged 18 to 80 years, scheduled for primary TKA for end-stage knee osteoarthritis, and able to provide consent. Exclusion criteria were preoperative hemoglobin (Hb) <110 g/L; TXA allergy; prior thromboembolic disease including deep vein thrombosis (DVT), pulmonary embolism (PE), stroke, myocardial infarction, or angina; traumatic or inflammatory arthritis; open orthopaedic surgery within 4 weeks; aspirin or other antiplatelet use within 1 week; and conditions requiring therapeutic or regular anticoagulation.

### Randomization and Blinding

A computer-generated block randomization sequence was used. Sequentially numbered opaque sealed envelopes were prepared by an independent investigator. On the day of surgery, 30 minutes before the procedure, an operating room staff member not involved in recruitment or outcome assessment opened the envelope and assigned patients to IV-TXA or IORA-TXA. Patients were blinded. Surgeons and operating room personnel were not blinded because administration routes differed. Outcomes were collected by a blinded investigator, and analyses were performed by a separate blinded researcher.

### Intervention and Surgery

Patients received TXA 20 mg/kg by either IV or IORA. In the IV-TXA group, TXA was given 30 minutes before skin incision. In the IORA-TXA group, the same dose was administered after tourniquet inflation and exposure of the femoral condyles and tibial plateau. The dose was divided equally and injected simultaneously into the medial femoral condyle and anteromedial proximal tibia, adapted from the study by Young et al.^7,8^. The IORA-TXA technique is illustrated in Fig. 1.

**Fig. 1:**
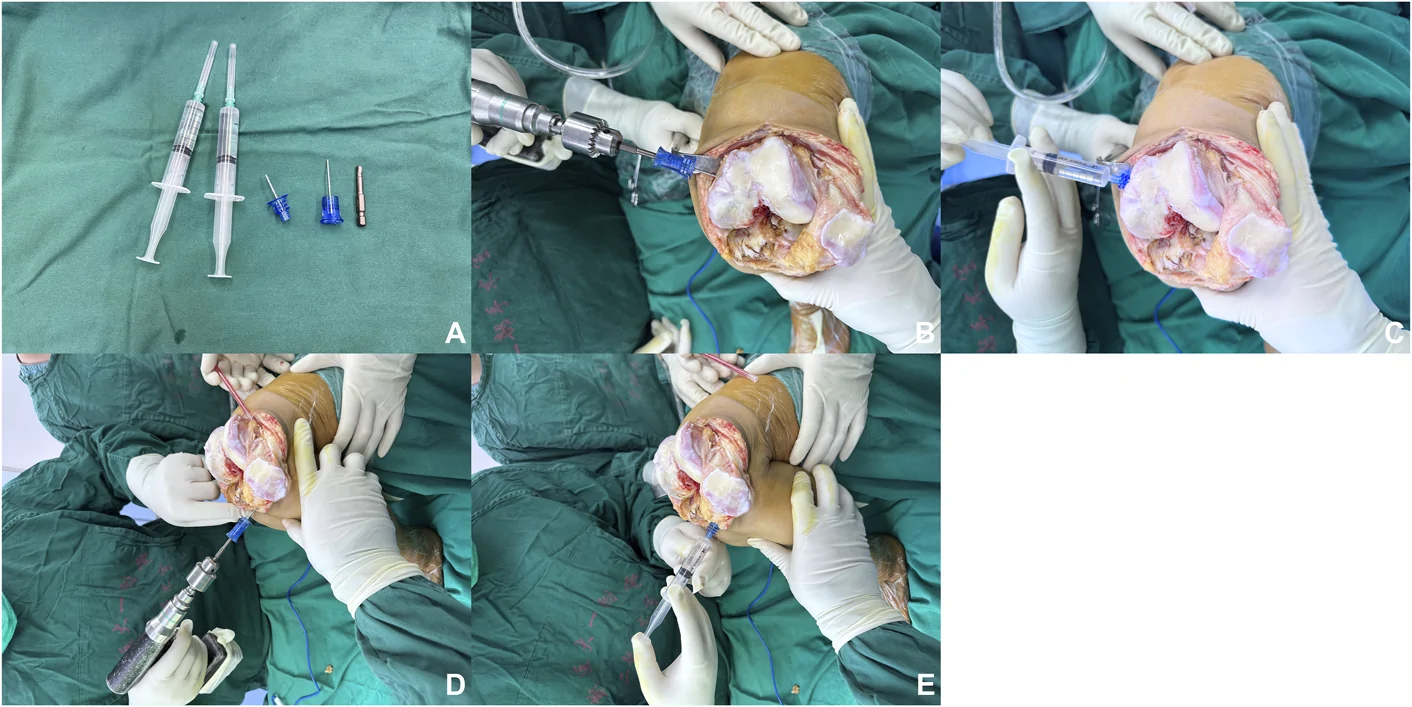
Images showing IORA-TXA during total knee arthroplasty. **Fig. 1-A** Preparation of 2 syringes containing the divided TXA dose and intraosseous access devices. **Fig. 1-B** Creation of the intraosseous entry site at the medial femoral condyle after tourniquet inflation and exposure of the distal femur and proximal tibia. **Fig. 1-C** Injection of TXA into the medial femoral condyle after confirmation of intraosseous needle placement. **Fig. 1-D** Creation of the intraosseous entry site at the anteromedial proximal tibia. **Fig. 1-E** Injection of TXA into the anteromedial proximal tibia. Successful intraosseous placement was confirmed clinically by a sudden decrease in resistance after cortical penetration and reduced resistance during injection; abnormal resistance or visible extravasation prompted needle adjustment or repositioning before completion of administration. IORA-TXA = intraosseous regional administration of tranexamic acid, and TXA = tranexamic acid.

Entry into the medullary cavity was confirmed by a sudden decrease in resistance after cortical penetration and reduced resistance during injection. Injection pressure was not quantitatively monitored with a transducer or manometer; instead, injection resistance was assessed manually. Increased resistance, failure of smooth injection, or visible extravasation suggested suboptimal needle position, prompting adjustment or repositioning before completion.

All operations were performed by a single senior surgeon (L.C.) and a fixed arthroplasty team. Spinal anesthesia was used in all patients. Mean arterial pressure was maintained within the clinically acceptable perioperative range; hypotension was treated with vasopressors and fluids according to institutional criteria. No intentional hypotensive anesthesia protocol was used. A tourniquet was inflated to 280 mm Hg before incision and released after wound closure. A standard medial parapatellar approach and cemented prostheses were used.

### Perioperative Management

All patients received cefuroxime 1,500 mg intravenously 30 minutes before incision; clindamycin 600 mg was used for cefuroxime allergy. Standardized periarticular analgesia was administered. No drains were used. Mechanical thromboprophylaxis and enhanced recovery measures included intermittent pneumatic compression, ankle-pump and quadriceps exercises, and ambulation as tolerated.

Pharmacologic thromboprophylaxis began 8 hours postoperatively with enoxaparin 2,000 IU, followed by enoxaparin 4,000 IU once daily from postoperative day 1 to day 2 and rivaroxaban 10 mg once daily from postoperative day 3 to day 14. Patients with Doppler-detected DVT or muscular calf vein thrombosis were evaluated by the surgical team and vascular medicine consultants and managed according to institutional anticoagulation protocols. Muscular calf vein thrombosis was recorded separately from DVT.

Transfusion followed AABB guidance: Hb < 80 g/L for all patients, including those with cardiovascular disease, with individualized decisions for symptomatic patients with Hb 80 to 100 g/L^18^.

## Outcomes

The primary outcome was calculated perioperative blood loss through postoperative day 3, estimated with the Gross formula^19^ using patient blood volume from the Nadler formula^20^. Hctpost was the lowest hematocrit on postoperative day 2 or 3 because hematocrit nadir after TKA commonly occurs after postoperative day 1 and these time points were routinely available before discharge.

Secondary efficacy outcomes were calculated hidden blood loss, postoperative Hb changes during the first 3 postoperative days, and transfusion rate. Calculated hidden blood loss was calculated as calculated perioperative blood loss minus intraoperative visible blood loss. Intraoperative visible blood loss was estimated from suction volume after subtraction of irrigation fluid and surgical field assessment.

Because the Gross and Nadler formulas provide estimates rather than direct measurements, calculated perioperative blood loss may be influenced by fluid shifts, hemodilution, hematocrit equilibration, transfusion status, and formula assumptions. Calculated hidden blood loss further compounds these limitations because it is a residual value after subtracting visually estimated visible loss.

Safety outcomes included DVT, muscular calf vein thrombosis, PE, wound-related complications, readmission, and anticoagulation-related adverse events. Bilateral lower extremity Doppler ultrasonography was performed on postoperative day 3. Wound complications, symptomatic venous thromboembolism, PE, readmission, and anticoagulation-related adverse events were assessed during hospitalization and at 4-week follow-up^21,22^.

### Sample Size Calculations

Pilot data showed calculated perioperative blood loss of 568.36 ± 292.26 mL with IORA-TXA and 763.60 ± 287.80 mL with IV-TXA, corresponding to Cohen d = 0.67. With G*Power 3.1, 44 patients per group provided 90% power at α = 0.05. Allowing 10% attrition, 49 patients per group were enrolled. The calculation was based on the primary efficacy outcome and was not powered for thromboembolic safety.

### Statistical Analysis

Analyses were performed using SPSS 25.0 and R 4.5.0. Two-sided P values of <0.05 were considered significant. Continuous variables were compared with independent samples *t* tests or Mann–Whitney U tests, as appropriate, and categorical variables were compared with chi-square or Fisher’s exact tests. Continuous outcomes are presented with between-group differences and 95% confidence intervals. For DVT outcomes, within-group 95% confidence intervals for incidence were calculated using exact binomial methods. Risk differences were reported descriptively as percentage point differences. Post hoc power for the observed DVT difference was exploratory.

## Results

Of the 110 patients assessed, 10 met exclusion criteria and 2 declined consent; 98 were analyzed, with 49 patients per group (Fig. 2). Baseline demographic and perioperative characteristics were balanced between groups, including sex, age, body mass index, operative side, operative time, tourniquet time, preoperative Hb, and preoperative hematocrit (Table 1). Women represented a substantial proportion of the cohort, consistent with the institutional TKA population and known predominance of advanced knee osteoarthritis among female patients; sex distribution was balanced between groups.

**Fig. 2:**
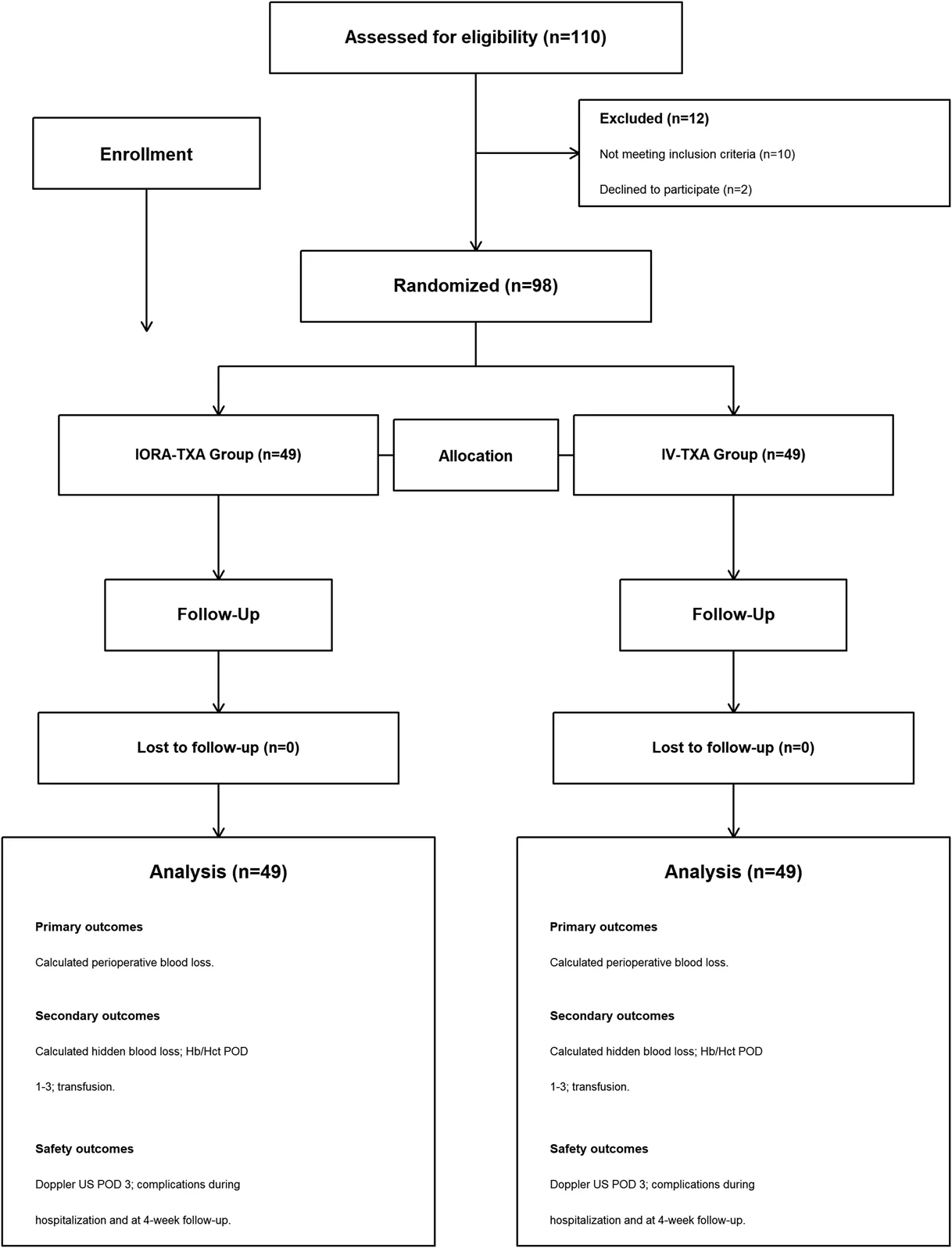
CONSORT flow diagram of the study design. CONSORT = Consolidated Standards of Reporting Trials.

**Table 1:** Baseline Characteristics of Patients

Demographics	IORA-TXA Group (n = 49)	IV-TXA Group (n = 49)	p
Year	65.4 ± 6.1	66.7 ± 6.5	0.163*
Sex (male/female)	7/42	8/41	1.000
Height (m)	1.61 ± 0.1	1.60 ± 0.1	0.758*
Weight (kg)	74.2 ± 10.2	73.2 ± 9.8	0.636*
BMI (kg/m^2^)	28.9 ± 4.2	28.2 ± 3.7	0.722*
ASA class			0.774
2	43 (87.8%)	41 (83.7%)	
3	6 (12.2%)	8 (16.3%)	
Comorbidities			
Hypertension	29 (59.2%)	31 (63.3%)	0.836
Diabetes	12 (24.5%)	8 (16.3%)	0.453
Coronary heart disease	6 (12.2%)	5 (10.2%)	1.000
Chronic bronchitis	3 (6.1%)	3 (6.1%)	1.000
Hepatitis	1 (2.0%)	0 (0.0%)	1.000
Chronic kidney disease	0 (0.0%)	0 (0.0%)	-
Preoperative laboratory values			
Hemoglobin (g/L)	134.6 ± 10.3	131.3 ± 11.3	0.130*
Hematocrit (%)	42 ± 3.2	41 ± 3.3	0.115*
Fibrinogen (g/L)	2.94 (2.62-3.19)	2.85 (2.56-3.05)	0.072†
Fibrin degradation product (mg/L)	1.00 (0.77-1.63)	1.13 (0.79-1.62)	0.835†
d-dimer (mg/L)	117.00 (83.00-177.00)	134.00 (82.00-197.00)	0.783†
CRP (mg/L)	3.62 (2.27-6.12)	2.81 (2.21-3.77)	0.107†
ESR (mm/h)	14.00 (10.00-20.00)	14.00 (10.00-18.00)	0.822†
LOS	10.00 (8.00-12.00)	10.00 (8.50-11.00)	0.505†
Operative time (min)	103.2 ± 26.4	111.0 ± 21.0	0.105*

ASA = American Society of Anesthesiologists, BMI = body mass index, CRP = C-reactive protein, ESR = erythrocyte sedimentation rate, IORA = Intraosseous Regional Administration, IV = intravenous, LOS = length of stay, and TXA = tranexamic acid.

Values are presented as mean ± standard deviation (SD), median (interquartile range [IQR]), or number (n). A two-sided p < 0.05 was considered statistically significant. Fisher's exact test was used for the comparison of categorical variables.

* The independent samples *t*-test.

† Mann–Whitney *U* test was used for the comparison of continuous variables.

IORA-TXA significantly reduced the primary outcome of calculated perioperative blood loss through postoperative day 3 compared with IV-TXA. Calculated hidden blood loss was significantly lower with IORA-TXA. Intraoperative visible blood loss values are summarized in Table 2. Postoperative Hb was similar between groups on postoperative day 1 but was significantly higher with IORA-TXA on postoperative days 2 and 3. No patient required transfusion. Detailed effect estimates, 95% CIs, and P values are summarized in Table 2.

**Table 2:** Efficacy Outcomes

Outcome	IORA-TXA, N = 49	IV-TXA, N = 49	Difference*	p
Calculated perioperative blood loss through postoperative day 3, mL	674.8 ± 335.0	817.5 ± 357.2	−142.6; 95% CI, −277.8 to −7.4	0.038
Calculated hidden blood loss, mL	644.8 ± 335.5	790.9 ± 339.0	−146.1; 95% CI, −281.3 to −10.9	0.035
Estimated intraoperative visible blood loss, mL	30.2 ± 14.4	26.5 ± 14.2	3.5, −2.3 to 9.2	0.234
Hb on postoperative day 1, g/L	134.0 ± 9.8	130.1 ± 11.1	3.9; 95% CI, −0.3 to 8.1	0.067
Hb on postoperative day 2, g/L	119.8 ± 8.3	113.8 ± 11.5	6.0; 95% CI, 2.0 to 10.0	0.005
Hb on postoperative day 3, g/L	114.8 ± 7.9	109.8 ± 12.6	5.0; 95% CI, 0.8 to 9.2	0.020
Blood transfusion, n (%)	0	0	-	-

CI = confidence interval, Hb = hemoglobin, IORA-TXA = intraosseous regional administration of tranexamic acid, and IV-TXA = intravenous tranexamic acid.

Values are presented as mean ± standard deviation unless otherwise indicated.

* Differences were calculated as IORA-TXA minus IV-TXA, with 95% confidence intervals for between-group differences. Negative values for blood loss outcomes indicate lower blood loss in the IORA-TXA group.

During the 4-week safety follow-up, no pulmonary embolism, wound-related complication, readmission for venous thromboembolism, or anticoagulation-related major bleeding occurred. Routine postoperative day 3 Doppler ultrasonography detected DVT in 5 patients in the IORA-TXA group and none in the IV-TXA group. These events were screen detected rather than symptom driven. All 5 DVTs were distal, resolved after standard anticoagulation, and were not associated with symptomatic PE or major bleeding during the 4-week follow-up. Muscular calf vein thrombosis occurred in 6 patients in the IORA-TXA group and 5 patients in the IV-TXA group. Event rates, exact binomial 95% CIs, risk differences, Fisher’s exact P values, and post hoc power are summarized in Table 3.

**Table 3:** Safety Outcomes

Safety Outcome	IORA-TXA, n (%)	95% CI,%*	IV-TXA, n (%)	95% CI,%*	Risk Difference, PP†	p
Doppler-detected deep vein thrombosis	5 (10.2%)	3.4 to 22.2	0	0 to 7.3	10.2	0.056
Muscular calf vein thrombosis	6 (12.2%)	4.6 to 24.8	5 (10.2%)	3.4 to 22.2	2.0	1.000
Pulmonary embolism	0	-	0	-	0	-
Wound-related complications	0	-	0	-	0	-
Readmission for venous thromboembolism	0	-	0	-	0	-
Anticoagulation-related major bleeding	0	-	0	-	0	-

CI = confidence interval, and pp = percentage points.

P values were calculated using Fisher’s exact test when applicable. P values were not calculated for outcomes with no events in either group. Post hoc power to detect the observed difference in Doppler-detected deep vein thrombosis was approximately 39% using an exact test framework.

* The 95% CIs are exact binomial confidence intervals for within-group incidence.

† Risk differences were calculated as IORA-TXA minus IV-TXA and are shown as percentage point differences.

## Discussion

To our knowledge, this is the first randomized trial of IORA-TXA in TKA. IORA-TXA reduced calculated perioperative blood loss and calculated hidden blood loss compared with single-dose IV-TXA. The absolute reduction in calculated perioperative blood loss was modest, and no transfusion difference was observed. However, in contemporary TKA cohorts with low transfusion rates, Hb preservation may be a more sensitive marker of blood management efficacy than transfusion alone. The higher Hb levels on postoperative days 2 and 3 therefore support a measurable early hematologic benefit. Thus, the findings demonstrate efficacy of IORA-TXA for reducing calculated perioperative blood loss compared with IV-TXA, while the higher DVT rate deserves further study.

The IV-TXA group had a calculated perioperative blood loss of 817.5 ± 357.2 mL, higher than in some single-dose IV studies^23,24^ and closer to values reported with split-dose protocols^25^. Cross-study variability likely reflects differences in dosing, calculation methods, tourniquet use, fluid management, timing of hematocrit measurement, and hidden loss estimation. Because blood loss end points were formula derived, they should be interpreted as estimates rather than direct measurements. The Gross formula may overestimate true blood loss compared with directly measured intraoperative loss, and formula-based blood loss methods can show poor intermethod agreement. Calculated hidden blood loss is further limited because it is derived as a residual after subtracting visually estimated visible blood loss from calculated perioperative blood loss.

Mechanistically, intraosseous delivery may increase regional drug exposure in cancellous bone, periosteum, synovium, capsule, and periarticular tissues^7-10,26-31^. Administration under tourniquet control may transiently sequester TXA in the operative limb, limiting early systemic dilution during bone resection, soft-tissue release, and tourniquet deflation, when local fibrinolytic activity may increase^32,33^. Preclinical and pharmacokinetic studies suggest that intraosseous TXA can achieve systemic exposure comparable with IV administration^13-16^, supporting biological plausibility for this route. Intraoperative visualization also allowed detection of extravasation and needle repositioning.

The DVT imbalance is clinically important. Five DVTs occurred in the IORA-TXA group versus none in the IV-TXA group, but the study was powered for calculated perioperative blood loss, not thromboembolic safety. Therefore, statistical nonsignificance should not be interpreted as evidence of safety equivalence^34^. All DVTs were detected by routine postoperative Doppler ultrasonography rather than symptoms alone. This distinction is important because routine screening can identify distal or clinically silent thromboses that may not be captured in studies relying only on symptomatic surveillance; nevertheless, screen-detected events remain relevant when evaluating a novel regional route of TXA administration.

Although pharmacologic prophylaxis continued to POD14 with rivaroxaban, approximating the minimum 10- to 14-day duration recommended by major guidelines, the early enoxaparin component was limited to the immediate postoperative period through POD2^35,36^. This brief early enoxaparin phase, together with POD3 Doppler screening, may have influenced detection of early, largely screen-detected DVTs. Although TXA is primarily antifibrinolytic rather than procoagulant and prior evidence suggests that IV or topical TXA does not increase clinically meaningful thromboembolism^3,4,6,37^, intraosseous pressurized infusion could theoretically affect marrow microvasculature, endothelial activation, or local coagulation^38,39^. This potential safety signal requires larger trials with predefined thromboembolic end points, standardized prophylaxis, and extended follow-up.

The comparator also limits interpretation. The IV group received a single 20-mg/kg dose 30 minutes before incision, consistent with the institutional protocol at trial design. However, contemporary practice increasingly uses multidose IV-TXA or combined IV plus topical strategies, which may provide stronger blood-sparing effects than single-dose IV-TXA^3,24,37,40^. Therefore, the observed benefit of IORA-TXA may not persist against optimized contemporary regimens. This limitation is central to interpreting comparative efficacy: This trial shows superiority over single-dose IV-TXA, but it does not establish superiority over current best practice TXA strategies. Future trials should compare IORA-TXA with optimized multidose or combined regimens rather than single-dose IV-TXA alone.

This trial has limitations. It was single-center, modest in size, and not powered for thromboembolic safety. TXA concentrations were not measured, limiting pharmacokinetic inference. Follow-up was 4 weeks, which may miss delayed events. Blood loss outcomes were based on formula-derived and residual-derived estimates vulnerable to hemodilution, fluid shifts, and visual estimation error. Routine tourniquet use and lack of surgeon blinding may limit generalizability and introduce performance bias, although outcome assessment and statistical analysis were blinded.

## Conclusions

IORA-TXA reduced calculated perioperative blood loss and calculated hidden blood loss and improved early Hb preservation compared with single-dose IV-TXA in TKA. However, the numerically higher DVT incidence represents an unresolved thromboembolic safety signal. Larger, adequately powered trials with standardized thromboprophylaxis, contemporary TXA comparators, predefined safety end points, and extended follow-up are needed.

## Funding Statement

This work was supported by research grants from the Natural Science Foundation of Xinjiang Uygur Autonomous (no. 2023D01E11) and the Major Special Projects of Science and Technology Plan of Xinjiang Uygur Autonomous Region (2022A03011).

## Ethical Review Statement

This trial was conducted in accordance with the Declaration of Helsinki and is reported in compliance with CONSORT guidelines. The study was prospectively registered (Chinese Clinical Trial Registry: CTR2400086474) and approved by our institutional ethics committee (240424-13); written informed consent was obtained from all participants.
